# The Dental Board of Victoria

**Published:** 1899-10

**Authors:** 


### The Dental Board of Victoria

Has framed regulations under the Dental Act, 1898, and forwarded them for the approval of the Governor in Council. Under those regulations, candidates for registration as dentists and for the diploma of Licentiate of Dental Surgery of Victoria must produce evidence of having passed a preliminary examination, served an apprenticeship, and pursued a course of professional study. The preliminary examination will be in English, German, French, Latin, arithmetic, algebra, geometry, history and geography, and candidates must pass in four of those subjects, including Latin. The apprenticeship must extend over a period of not less than three years, and be served with a registered Victorian dentist. The course of professional study must extend over four years, the subjects of study being chemistry, anatomy, dental mechanics and metallurgy, materia medica and therapeutics, physiology and histology, general surgery and pathology, dissections, medicine, dental anatomy and oral surgery, dental surgery, pathology and bacteriology. Four examinations must also be passed before the Board.—*Pharmaceutical Journal.*

